# A NATIONWIDE EXAMINATION OF MEDICARE PART B UTILIZATION DURING HOSPICE ELECTION

**DOI:** 10.1093/geroni/igac059.159

**Published:** 2022-12-20

**Authors:** Thomas Christian, Michael Plotzke

**Affiliations:** Abt Associates, Cambridge, Massachusetts, United States; Abt Associates, Saint Louis, Missouri, United States

## Abstract

This research characterizes trends in two hospice-specific modifiers: (1) “GV” indicating services for the terminal/related conditions by an attending physician not an employee of the hospice and (2) “GW” indicating physician services unrelated to terminal/related conditions. We identified Part B (carrier/physician supplier) claims during hospice elections in Federal Fiscal Year (FY)2020, and replicated an Office of Inspector General (OIG) approach calculating potentially “questionable” Part B claims, where the physician and diagnosis codes match between the hospice and Part B claims (without a GW modifier listed). Using logistic regression, we calculated adjusted odds ratio (AOR) and 95% confidence intervals (CI) to characterize this billing. Overall, $372.8 million in physician services occurred during hospice elections in FY2020. Of this, two-thirds ($247.8 million) included a GW modifier, one-quarter ($86.5 million) a GV modifier, $2.2 million both modifiers, and $40.8 million neither modifier. Replicating the OIG methodology, we calculated $19.4 million (5.2%) as “questionable”. Beneficiaries electing hospice for 180+ days were three times more likely (95% CI 2.99-3.12) to have questionable billing as a beneficiary electing hospice 14-29 days, and facility residents were more likely to have questionable billing, relative to beneficiaries in their own homes. Questionable billing rates were also highest in the northeastern quadrant of the country. Lastly, we found ten percent of physicians accounted for almost three-quarters of all questionable billing. CMS should further monitor physician services during hospice to maintain the integrity of the benefit and ensure beneficiaries receive adequate care.

